# Research on statistical measure under double carbon target - Self-moving regression model of grey prediction based on entropy weight method

**DOI:** 10.12688/f1000research.162696.2

**Published:** 2025-05-06

**Authors:** Sanglin Zhao, Hao Deng, Jackon Steve

**Affiliations:** 1 School of Engineering Management, Hunan University of Finance and Economics, Changsha, Hunan, China; 2 School of Management, University of Khartoum, Khartoum, Khartoum, Sudan

**Keywords:** Grey Prediction; Ridge Regression; Entropy Weight Method; ARIMA Model; Kaya Model

## Abstract

**Background:**

At the 2020 UN General Assembly, China pledged to peak carbon emissions before 2030 and achieve carbon neutrality by 2060. However, the traditional social development model has led to increasing carbon emissions annually, highlighting the need to resolve the contradiction between development and carbon reduction. This study examines the relationship between carbon emissions, economy, population, and energy consumption in a specific region to support carbon peak and neutrality goals.

**Methods:**

A comprehensive indicator system was established, encompassing economic, population, energy consumption, and carbon emissions indicators. The study analyzed these factors during the 12th and 13th Five Year Plans, comparing total carbon emissions in 2010 and across the plans, and assessing trends. It also comprehensively analyzed the relationships and mutual influences among these factors. The study identified the main challenges in achieving carbon peak and neutrality. Using the Kaya model and various factor models, it calculated carbon peak times for three scenarios: baseline (2022), natural (2036), and ambitious (2021). These findings provide a basis for dual carbon path planning.

**Result:**

The research results indicate that carbon emissions are closely related to the economy, population, and energy consumption. The prediction shows that the future trend of carbon emissions is controllable. Suggestions for dual carbon path planning are proposed to provide empirical basis for policy formulation. Under the baseline scenario, the peak carbon emissions are expected to occur around 2022; Under natural circumstances, the peak carbon emissions will be postponed to 2036; In the ambitious scenario, the carbon peak time can be advanced to 2021.

**Conclusion:**

The research results are crucial for achieving carbon reduction targets and sustainable development and can be used to formulate targeted policies to promote regional development and support China’s carbon neutrality commitments.

## 1. Introduction

### 1.1 Research background

With the rapid progress of industrialization and urbanization, global carbon emissions continue to increase, which leads to the increasingly serious problem of climate change. The report issued by the United Nations Intergovernmental Panel on Climate Change (IPCC) points out that since the industrial revolution, the global temperature has risen significantly, and extreme weather events have occurred frequently, posing a serious threat to human society and natural ecosystems. In this context, achieving peak carbon dioxide emissions and carbon neutrality has become the common goal of all countries in the world.

As the largest energy consumer and carbon emitter in the world, China’s energy consumption structure, carbon emission characteristics and economic growth model have an important impact on global climate change. Therefore, China’s goal of double carbon is not only a requirement for its own development, but also a contribution to the global response to climate change.

Although China has shown firm determination and strong action to achieve the goal of double carbon, it still faces many challenges and difficulties in the actual process. First of all, the transformation of energy structure is the key link to achieve the goal of double carbon, but at present, China’s energy consumption is still dominated by coal, and the proportion of clean energy is relatively low, so the transformation of energy structure is under great pressure. Secondly, technological innovation and popularization are the important support to achieve the goal of double carbon, but there is still a certain gap in clean energy technology, carbon capture and storage technology in China. In addition, policies and governance, social and economic transformation and other aspects also need to make great efforts.

***Importance of Statistical Measure Research***

Under the guidance of the dual-carbon target, how to scientifically and accurately evaluate carbon emissions, analyze the relationship between carbon emissions and economy, population and energy consumption, and predict the future carbon emission trend has become an urgent problem. As an important analytical tool and method, statistical measurement research can provide scientific basis and decision support for formulating targeted policies and measures. Through in-depth analysis of the relationship between carbon emissions and economy, population and energy consumption, as well as the challenges faced by peak carbon dioxide emissions to achieve carbon neutrality, more scientific and reasonable policies and measures can be formulated to promote regional sustainable development and achieve carbon emission reduction targets.

In this study, the grey prediction self-moving regression model based on entropy weight method is used to predict and analyze the carbon emissions in a certain area. As an objective weighting method, entropy weight method can determine the weight of indicators according to the degree of correlation between indicators and the size of information entropy; The grey forecasting model is suitable for forecasting problems with less data and incomplete information. The self-moving regression model can capture the trend and periodicity of time series through the analysis of historical data. By combining these three methods, we can evaluate and analyze the carbon emission situation in this region more comprehensively and accurately, and predict the future carbon emission trend, which provides an important basis for formulating the dual-carbon path planning.

### 1.2 Research significance

With the increasingly serious problem of global climate change, our government has actively responded to the voice of the international community and put forward the goal of double carbon, namely, peak carbon dioxide emissions and carbon neutrality. This goal means that China should achieve the peak of carbon emissions within the specified time, and gradually reduce carbon emissions in the subsequent time to finally achieve carbon neutrality. In order to achieve this goal better, this study aims to analyze the relationship between carbon emissions and economy, population and energy consumption through in-depth statistical measurement research, so as to provide scientific basis for formulating targeted policies and measures.

This study adopts a grey prediction autoregressive model based on entropy weight method to accurately predict and analyze carbon emissions in a certain region, providing an important foundation for dual carbon path planning. By predicting carbon emissions, we gain a clearer understanding of future trends and formulate targeted policies to promote the achievement of carbon peak and carbon neutrality goals.

In the process of research, we first theoretically analyzed the relationship between carbon emissions and economic, population and energy consumption, and made clear the contribution degree of each factor to carbon emissions. Then, we use the self-moving regression model of grey prediction based on entropy weight method to make an empirical analysis and calculation of carbon emissions in Jiangsu Province. By setting and optimizing the model parameters, we get more accurate carbon emission prediction results.

According to our research, we find that there are many challenges to achieve peak carbon dioxide emissions and carbon neutrality. First of all, the rapid development of China’s economy has brought about the rapid growth of carbon emissions, and it takes great efforts to achieve the peak of carbon emissions in a short time. Secondly, China’s energy consumption structure is dominated by fossil energy, which aggravates the carbon emission problem to some extent. Therefore, in the process of achieving the goal of double carbon, we need to actively promote the optimization of energy consumption structure and increase the proportion of clean energy.

According to our forecast and analysis, we provide targeted policy suggestions for government departments. On the one hand, we should strengthen the control of carbon emissions, promote the adjustment of industrial structure, improve energy efficiency and reduce the intensity of carbon emissions per unit GDP. On the other hand, it is necessary to increase R&D and investment in clean energy and promote the transformation of energy consumption structure. In addition, international cooperation should be strengthened to jointly address global climate change.

Based on the research on the relationship between carbon emissions and economic, population and energy consumption, this study provides beneficial enlightenment for China to achieve the goal of peak carbon dioxide emissions and carbon neutrality. We firmly believe that with the active promotion of government departments, China will be able to successfully achieve the goal of double carbon and make positive contributions to the response to global climate change.

### 1.3 Literature review

In recent years, the research on double carbon targets in China has been deepened, involving a wide range of fields and diverse research methods. In the research of statistical measurement of carbon emissions, domestic scholars have deeply discussed the changing trend, influencing factors and the relationship with economy, population and energy consumption based on different theoretical frameworks and model methods.

On the one hand, scholars have comprehensively analyzed the influencing factors of carbon emissions by establishing a comprehensive index system including economic indicators, population indicators, energy consumption indicators and carbon emissions indicators. Use a variety of statistical methods and models, such as multiple linear regression, grey prediction, entropy weight method, etc. ^1– 6^ Combined with Kaya model ^7^ The changing trend of carbon emissions is predicted and analyzed, which provides an important basis for formulating the dual-carbon path planning.

On the other hand, scholars are also concerned about the challenges and difficulties in realizing peak carbon dioxide emissions and carbon neutrality, especially the transformation of energy structure, technological innovation and promotion, policy and governance, and socio-economic transformation. Through in-depth study of the status quo and problems in these fields, the corresponding policies and suggestions are put forward to promote the realization of China’s double-carbon goal.

In addition, there are some studies that focus on the carbon emissions of specific industries or regions. For example, the carbon emissions of large carbon emitters such as power industry and transportation industry are deeply studied, and their emission characteristics and influencing factors are analyzed, and corresponding emission reduction measures and suggestions are put forward. ^8, 9^ At the same time, some regions have also carried out the exploration and practice of carbon market mechanisms such as carbon emission trading and carbon sink, which provides useful experience and reference for the realization of China’s dual-carbon goal. ^10^

Internationally, the goal of double carbon has also become the focus of global attention. Governments and scholars all over the world have carried out relevant research to promote the process of global response to climate change.

In the research of statistical measurement of carbon emissions, foreign scholars have also adopted a variety of methods and models to study. They not only pay attention to the changing trend and influencing factors of carbon emissions, but also pay attention to the relationship between carbon emissions and economic development and social well-being. By establishing a global or regional carbon emission database, the global or regional carbon emission situation is comprehensively analyzed and compared, which provides data support for the international community’s emission reduction actions. ^11– 18^

At the same time, foreign scholars are also concerned about the technological and institutional innovations needed to achieve carbon neutrality. They have provided technical support and institutional guarantee for global emission reduction actions through in-depth study of cutting-edge technologies such as renewable energy technology, carbon capture and storage technology, smart grid, and market mechanisms such as carbon emission trading and carbon tax.

In addition, some international organizations and non-governmental organizations also actively carry out research and advocacy work under the goal of double carbon. For example, the International Energy Agency (IEA) regularly issues global carbon emissions reports to analyze the current situation and trends of global carbon emissions; The United Nations Environment Programme (UNEP) actively promotes global cooperation and actions to address climate change. ^19– 24^

To sum up, the research under the dual-carbon target at home and abroad has been deepened and expanded, which has provided strong support and promotion for the global action to deal with climate change.

## 2. Methods

### 2.1 Index and model construction

#### Building objectives and data sources

1. **Build goals**
  (1) Establish indicators and indicators system
  This study establishes an index system that can describe economy, population, energy consumption and carbon emissions. This includes economic indicators (such as GDP and economic growth rate), population indicators (such as total population and population growth rate), energy consumption indicators (such as total energy consumption and energy consumption structure) and carbon emission indicators (such as total carbon emission and carbon emission intensity).
  (2) Current situation of regional carbon emissions
  Analyze the current situation of carbon emissions, economy, population and energy consumption in different periods (such as the Twelfth Five-Year Plan and the Thirteenth Five-Year Plan). Specific analysis includes:
  ① Compare the total carbon emissions in 2010, the Twelfth Five-Year Plan and the Thirteenth Five-Year Plan, and analyze their changing trends.
  ② This paper analyzes the factors that have an impact on carbon emissions in this region, such as economic growth, population growth and changes in energy consumption structure, and evaluates their contributions to carbon emissions.
  (3) The main challenges to realize peak carbon dioxide emissions and carbon neutrality
  The main challenges faced by Jiangsu Province in realizing peak carbon dioxide emissions and carbon neutrality are analyzed. This may include:
  ① Transformation of energy structure: how to reduce dependence on fossil fuels and increase the proportion of non-fossil energy.
  ② Technological innovation and popularization: how to promote the research, development, application and popularization of low-carbon technologies, including energy efficiency improvement and clean energy utilization.
  ③ Policy and governance: how to establish and improve carbon market mechanism, energy policy, environmental supervision, etc. to promote the realization of carbon emission reduction targets.
  ④ Socio-economic transformation: how to achieve carbon emission reduction while ensuring economic development, employment and social stability.
  (4) Each index and its correlation model
  It is necessary to analyze the changes of relevant indicators and establish the correlation model between indicators in order to predict the changes of carbon emissions. It needs to be related to the determination of parameters such as the improvement of energy utilization efficiency and the proportion of non-fossil energy consumption, and at the same time take into account the multiple effects of double carbon policy and technological progress.
  To sum up, it is necessary to establish an index system, analyze the current situation and challenges, and establish a correlation model, so as to fully understand the regional carbon emissions and economic, population and energy consumption, and provide a basis for the dual-carbon path planning.
2. **Data sources**
  In order to ensure the authoritative source of data, the energy consumption data in this paper comes from IEA ( CO2 Emissions in 2023–Analysis - IEA, https://www.iea.org/reports/co2-emissions-in-2023). ^25^

### 2.2 Indicators and indicator system

In this paper, according to the four indicators of economy, population, energy consumption and carbon emissions, the index system of influencing factors of double carbon target is constructed, and the specific indicators are as follows.

### 2.3 Status of Regional Carbon Emissions

Requirement 1: The indicators can describe the economy, population, energy consumption and carbon emissions of a certain region;

Requirement 2: Analyze the factors that affect the carbon emissions in this region and their contributions;

Requirement 3: Judge the main challenges that the region needs to face to achieve peak carbon dioxide emissions and carbon neutrality, and provide the basis for the differentiated path selection in the regional dual-carbon (peak carbon dioxide emissions and carbon neutrality) path planning.

During the period of 2010-2015, the carbon emission fluctuated relatively with time, and the overall trend was that it first increased and then decreased. During this period, it can be well fitted by quadratic function. During the period of 2015-2020, the fluctuation of carbon emissions with time is relatively small, and the overall trend is increasing. In 2020, carbon emissions will decrease. During this period, a straight line can be used for fitting (during the whole period (2010-2020), a straight line can be used for fitting: $R^{2}=0.7605)$

y=1273.2x−2E+6 (1)

The descriptive statistical analysis of carbon emission data by SPSSPRO shows that the data variance is large, so the carbon emission fluctuates greatly from 2010 to 2020.

To analyze the factors that have an impact on carbon emissions in this region, such as economic growth, population growth, changes in energy consumption structure, etc., and evaluate the contribution to carbon emissions. We use Pearson correlation test, which can effectively test and analyze the influencing factors of carbon emissions.

Using SPSSPRO to test the normality, the following Table 3 and Tables 1- 5 is obtained:

**Table 1.** Index selection.

Primary index	Secondary index	Index definition	Number	Unit	Indicator direction
economic indicator	GDP	GNP	*X*1X1	%	+
	GDP growth rate	(GDP of the following year-GDP of the previous year)/GDP of the previous year	*X*2X2	%	+
	Industrial added value	Industrial added value	*X*3X3	%	-
	The tertiary industry accounted for	Gross value of tertiary industry/GDP	*X*4X4	%	+
human population	Total population	resident population	*X*5X5	%	+
	population rate of increase	(Population of the year after-population of the year before)/population of the year before.	*X*6X6	%	+
Energy consumption index	Proportion of coal consumption	Coal consumption/total energy consumption	*X*7X7	%	-
Carbon emission index	Total carbon emissions	Total carbon emissions	*X*8X8	%	-
	Carbon emission intensity	Total carbon emissions/GDP	*X*9X9	%	-
	Per capita carbon emissions	Total carbon emissions/total population	*X*10X10	%	-

Note: The direction of indicators is relative to carbon emissions.

**Table 2.** Carbon emission fitting data.

variable name	Sample size	maximum	minimum value	average value	standard deviation
Carbon emissions	11	74096.331	56360.052	67631.164	4842.24

variable name	median	variance	kurtosis	skewness	Coefficient of variation (CV)
Carbon emissions	67502.613	23447287.915	2.136	-1.072	0.072

**Table 3.** Normality test result.

variable name	Sample size	median	average value	standard deviation	skewness	kurtosis	S-W test	K-S test
Residents' living consumption	11	5721.341	5940.981	1155.787	0.171	-1.49	0.926(0.374)	0.149(0.938)
Construction consumption department	11	3944.317	4226.376	944.741	0.318	-1.109	0.944(0.571)	0.163(0.888)
Traffic consumption department	11	4398.073	4338.79	836.924	-0.094	-1.247	0.949(0.633)	0.099(0.999)
Total carbon emission	11	67502.613	67631.164	4842.24	-1.072	2.136	0.92(0.320)	0.192(0.744)
Industrial consumption sector	11	52506.881	51983.922	2445.754	-2.394	6.767	0.722(0.001***)	0.268(0.344)
Agriculture and forestry consumption department	11	1165.275	1141.095	131.678	-0.614	-0.853	0.91(0.246)	0.201(0.696)

Note: * * *, * * and * represent the significance levels of 1%, 5% and 10% respectively.

**Table 4.** Pearson correlation test analysis.

	Residents' living consumption	Construction consumption department	Agriculture and forestry consumption department	Traffic consumption department	Total carbon emission	Industrial consumption sector
Residents' living consumption	1(0.000***)	0.99(0.000***)	0.865(0.001***)	0.955(0.000***)	0.895(0.000***)	0.544(0.083*)
Construction consumption department	0.99(0.000***)	1(0.000***)	0.869(0.001***)	0.958(0.000***)	0.916(0.000***)	0.585(0.059*)
Agriculture and forestry consumption department	0.865(0.001***)	0.869(0.001***)	1(0.000***)	0.859(0.001***)	0.906(0.000***)	0.701(0.016**)
Traffic consumption department	0.955(0.000***)	0.958(0.000***)	0.859(0.001***)	1(0.000***)	0.869(0.001***)	0.512(0.108)
Total carbon emission	0.895(0.000***)	0.916(0.000***)	0.906(0.000***)	0.869(0.001***)	1(0.000***)	0.857(0.001***)
Industrial consumption sector	0.544(0.083*)	0.585(0.059*)	0.701(0.016**)	0.512(0.108)	0.857(0.001***)	1(0.000***)
Note: * * *, * * and * represent the significance levels of 1%, 5% and 10% respectively.						

Department	Contribution rate
Agriculture and forestry consumption department	0.016872328
Construction consumption department	0.062491552
Residents' living consumption	0.087843837
Traffic consumption department	0.064153709
Industrial consumption sector	0.768638589

**Table 5.** Trend of index change.

variable name	Sample size	maximum	minimum value	average value	standard deviation	median	variance	kurtosis	skewness	Coefficient of variation (CV)
GDP growth rate	10	0.11	0.037	0.079	0.022	0.082	0	0.18	-0.544	0.278
Industrial added value	10	7750.09	-1819.197	772.835	2717.918	253.759	7387076.052	5.45	2.117	3.517
The tertiary industry accounted for	10	0.525	0.424	0.479	0.034	0.485	0.001	-1.138	-0.34	0.072
population rate of increase	10	0.02	0.001	0.007	0.006	0.006	0	1.009	1.038	0.752
Proportion of energy consumption coal	10	0.904	0.8	0.85	0.038	0.858	0.001	-1.467	-0.139	0.045
Total carbon emissions	10	74096.331	64853.276	68758.276	3244.33	68014.369	10525677.865	-1.252	0.413	0.047
Carbon emission intensity	10	1.419	0.819	1.05	0.204	0.989	0.042	-0.558	0.817	0.195
Per capita carbon emissions	10	8.749	7.831	8.269	0.281	8.245	0.079	-0.438	0.149	0.034
GDP	10	88683.215	45952.65	67958.936	14806.636	68108.853	219236460.368	-1.331	-0.052	0.218
resident population	10	8477.26	8022.99	8312.895	157.399	8348.29	24774.32	-0.613	-0.75	0.019

The results of normality test based on S-W test or K-S test are as follows:

For the samples of residents’ living consumption, construction consumption sector, transportation consumption sector, total carbon emission and agriculture and forestry consumption sector, the sample number n is less than 5000, so S-W test is adopted.

The results show that the significance p values of these samples are 0.374, 0.571, 0.633, 0.320 and 0.246, respectively, which are not significant, so the original hypothesis can not be rejected, that is, the data of these samples meet normal distribution.

However, for the sample of industrial consumption department, its significance P value is 0.001, which shows the significance level, so the original hypothesis is rejected and the data of this sample does not meet the normal distribution.

It can be seen that Pearson correlation test analysis can be carried out in the next step.

The table shows the parameter result table of model test, including correlation coefficient and significance P value.

1. First, test whether there is a statistically significant relationship between XY, and judge whether the P value is significant (P<0.05).
2. If it is significant, it means that there is correlation between the two variables, otherwise, there is no correlation between the two variables.
3. Analyze the positive and negative directions of the correlation coefficient and the degree of correlation.

From the thermodynamic coefficient diagram formed by the matrix of influencing factors, it can be seen that the main factors affecting carbon emissions are agriculture and forestry consumption departments (0.906), construction consumption departments (0.916), residents’ living consumption (0.895), followed by transportation consumption departments (0.869) and industrial consumption departments (0.857).

$$\text{Define contribution rate}=\frac{\sum_{2010}^{2020}\text{Annual carbon emissions of}\;a\;\text{certain department}}{\text{Total carbon emissions}}\in \left[0,1\right]$$

### 2.4 Regional carbon emission indicators and their related models

1. **Trends of indicators**
2. **Correlation analysis**
  Entropy weight method is an objective way of empowerment, and its core lies in determining the corresponding entropy weight according to the information provided by each index for decision makers. Relatively speaking, the grey relational analysis method focuses on evaluating the degree of correlation between things and factors by considering the similarity or difference of development trends among factors, especially good at dynamic analysis. In view of the fact that the amount of index data in actual situations is often limited and the degree of variation of index data is uncertain, in order to improve the objectivity of the evaluation index system, this paper uses the method of combining entropy weight with grey relational analysis to make a time dynamic evaluation of regional carbon emissions in China. The following are the specific calculation steps of entropy weight-gray correlation method.

(1) Standardize and calculate the specific gravity of index value

Firstly, the index is dimensionless.

Positive indicators:

$$x_{\mathrm{ij}}^{\prime }=\frac{x_{\mathrm{ij}}-\min\left(x_{\mathrm{ij}}\right)}{\max\left(x_{\mathrm{ij}}\right)-\min\left(x_{\mathrm{ij}}\right)}$$ (2)

Negative indicator:

$$x_{\mathrm{ij}}^{\prime }=\frac{\max\left(x_{\mathrm{ij}}\right)-x_{\mathrm{ij}}}{\max\left(x_{\mathrm{ij}}\right)-\min\left(x_{\mathrm{ij}}\right)}$$ (3)

Then, calculate the index value proportion:

$$y_{\mathrm{ij}}=\frac{x_{\mathrm{ij}}^{\prime }}{\sum_{i=1}^{m}x_{\mathrm{ij}}^{\prime }\left(0\leq y_{\mathrm{ij}}\leq 1\right)}$$ (4)

(2)-(4) Where: is the numerical value of the j-th index in the ith year, is the standardized value, is the minimum value of the j-th index, is the maximum value of the j-th index, and is the index proportion $x_{\mathrm{ij}}x_{\mathrm{ij}}^{\prime }\min\left(x_{\mathrm{ij}}\right)\max\left(x_{\mathrm{ij}}\right)y_{\mathrm{ij}}$

(2) Entropy weight method to find the weight.

Calculation of information entropy value and information utility value; $e_{j}d_{j}$

$$e_{j}=-K\sum_{i=1}^{m}y_{\mathrm{ij}}\ln\left(y_{\mathrm{ij}}\right)$$ (5)

$$d_{j}=1-e_{j}=1+K\sum_{i=1}^{m}y_{\mathrm{ij}}\ln\left(y_{\mathrm{ij}}\right)$$ (6)

In the above formula, it is the information entropy of the j-th index; M is the number of observed values; K=, which is a constant; Is the information utility value $e_{j}\frac{1}{\;\mathrm{lnm}}d_{j}$

Finally, calculate the weight of each index.

$$W_{j}=\frac{d_{j}}{\sum_{i=1}^{n}d_{j}}$$ (7)

The above formula: is the weight of j indicators, and n is the number of indicators $W_{j}$

The weight analysis and calculation results of entropy weight method are shown in Table 6 below.

**Table 6.** Weight analysis and calculation results of entropy weight method.

Entropy weight method			
item	Information entropy value e	Information utility value d	Weight (%)
GDP	0.889	0.111	12.979
GDP growth rate	0.929	0.071	8.292
The tertiary industry accounted for	0.899	0.101	11.887
resident population	0.92	0.08	9.448
Industrial added value	0.949	0.051	5.961
population rate of increase	0.939	0.061	7.148
Proportion of energy consumption coal	0.889	0.111	12.988
Total carbon emissions	0.905	0.095	11.196
Carbon emission intensity	0.915	0.085	10.013
Per capita carbon emissions	0.914	0.086	10.087

The chart shows that the weight of GDP is 12.979%, the weight of GDP growth rate is 8.292%, the weight of tertiary industry is 11.887%, the weight of resident population is 9.448%, the weight of industrial added value is 5.961%, the weight of population growth rate is 7.148%, the weight of energy consumption coal is 12.988%, and the weight of total carbon emissions is 11.11. The weight of carbon emission intensity is 10.013%, and the weight of per capita carbon emission is 10.087%, in which the maximum index weight is the proportion of energy consumption coal (12.988%) and the minimum index industrial added value (5.961%).

(3) The grey correlation method is used to calculate the correlation degree.

First, a reference sequence and a comparison sequence are determined. The reference sequence is:

$$x_{i}=\left(x_{i1},x_{i2},x_{i3},\ldots \ldots 。x_{\text{in}}\right)\left(i=1,2,\ldots \ldots ,\;m\right)$$

The comparison sequence consists of the optimal values of each index, because the dimensionless indexes belong to the interval [0,1]. Therefore, the maximum value of each index is selected as the comparison sequence =(1,1, …, 1). $x_{0}$

Secondly, calculate the correlation coefficient:

$$\varepsilon_{\mathrm{ij}}=\frac{\min_{i}\min_{j}\left|x_{\mathrm{ij}}-1\right|+\rho \max_{i}\max_{j}\left|x_{\mathrm{ij}}-1\right|}{\left|x_{\mathrm{ij}}-1\right|+\rho \max_{i}\max_{j}\left|y_{\mathrm{ij}}-1\right|}\left(i=1,2,\ldots \ldots ,\;m;j=1,2,\ldots \ldots ,n\right)$$ (8)

In the above formula, ρ is the resolution coefficient, and 0≤ρ≤1. Generally, when ρ=0.5, it has higher resolution. (Resolution coefficient ρ∈(0, ∞), the smaller the ρ, the greater the resolution. Generally, the value range of ρ is (0,1), and the specific value can be determined according to the situation. When ρ≤0.5463, the resolution is the best, usually ρ=0.5).

(4) Weighted correlation degree between reference sequence and comparison sequence

$$r_{i}=\sum_{j=1}^{n}w_{j}\varepsilon_{\mathrm{ij}}\left(i=1,2,\ldots \ldots ,m\right)$$

Where: is the entropy weight. The correlation degree reflects the closeness between the evaluation object and the optimal state, and the greater the value, the higher the closeness between the I-th evaluation object and the optimal state. Therefore, the evaluation objects can be sorted and classified according to their relevance $w_{j}r_{i}r_{i}$

The calculation results are shown in Table 7 and Tables 8- 15 below:

**Table 7.** Correlation degree result.

Evaluation item	Degree of association	Ranking
Per capita carbon emissions	0.997	one
Resident population	0.994	2
The tertiary industry accounted for	0.993	three
Proportion of energy consumption coal	0.983	four
GDP	0.97	five
Carbon emission intensity	0.961	six
GDP growth rate	0.948	seven
Population rate of increase	0.886	eight
Industrial added value	0.728	nine

**Table 8.** Linear regression calculation.

The result of linear regression analysis is n=11.								
	Non-Standardized coefficient	Standardization coefficient	t	P	VIF	R ^2^	Adjust r	F
	standard error	Beta						
Constant	0	-	-0.383	0.712	-	one	one	F=9.573441859447495e+28 P=0.000***
GDP	0	one	124302039680495.56	0.000***	12.392			
Resident population	0	0	0.35	0.735	12.392			
Dependent variable: total energy consumption								

Note: * * *, * * and * represent the significance levels of 1%, 5% and 10% respectively.

**Table 9.** Ridge regression calculation results.

K=0.03	Non-standardized coefficient		Standardization coefficient	t	P	R ^2^	Adjust r	F
	B	Standard error	Beta					
Constant	-121268.079	27901.567	-	-4.346	0.002***	0.996	0.995	1026.901(0.000***)
Resident population	16.388	3.689	0.203	4.442	0.002***			
GDP	0.782	0.046	0.782	17.096	0.000***			
Dependent variable: total energy consumption								

Note: * * *, * * and * represent the significance levels of 1%, 5% and 10% respectively.

**Table 10.** Forecast results of resident population.

t	2020	2021	2022	2023	2024	2025	2026	2027	2028	2029	2030
resident population	8477.26	8619.249	8677.118	8734.987	8792.856	8850.725	8908.594	8966.463	9024.332	9082.201	9140.07
t	2030	2031	2032	2033	2034	2035	2036	2037	2038	2039	2040
resident population	9140.07	9197.939	9255.808	9313.677	9371.546	9429.415	9487.284	9545.153	9603.022	9660.891	9718.76
t	2041	2042	2043	2044	2045	2046	2047	2048	2049	2050	
resident population	9776.629	9834.498	9892.367	9950.236	10008.11	10065.97	10123.84	10181.71	10239.58	10297.45	

**Table 11.** GDP forecast results.

t	GDP	t	GDP	t	GDP
2020	88683.214	2031	143503.836	2041	192236.836
2021	94770.836	2032	148377.136	2042	197110.136
2022	99644.136	2033	153250.436	2043	201983.436
2023	104517.436	2034	158123.736	2044	206856.736
2024	109390.736	2035	162997.036	2045	211730.036
2025	114264.036	2036	167870.336	2046	216603.336
2026	119137.336	2037	172743.636	2047	221476.636
2027	124010.636	2038	177616.936	2048	226349.936
2028	128883.936	2039	182490.236	2049	231223.236
2029	133757.236	2040	187363.536	2050	236096.536
2030	138630.536				

**Table 12.** Regional carbon emission prediction model.

The result of linear regression analysis is n=11.									
	Non-standardized coefficient		Standardization coefficient	t	P	VIF	R ^2^	Adjust r	F
	B	Standard error	Beta						
constant	-71496.169	101547.402	-	-0.704	0.501	-	0.797	0.746	F=15.708 P=0.002***
GDP	0.034	0.084	0.113	0.403	0.698				
resident population	16.282	13.544	0.674	1.202	0.264	12.392			
Total energy consumption	0.034	0.084	0.113	0.403	0.698				
Dependent variable: total carbon emissions									

Note: * * *, * * and * represent the significance levels of 1%, 5% and 10% respectively.

**Table 13.** Variable definition.

variable	Variable interpretation
C	Total carbon emission
P	Total population
G	Gross GDP
E	Total energy consumption
g	Per capita GDP
e	Energy intensity, energy consumed per unit GDP
c	Energy carbon emission intensity, carbon emission per unit energy consumption
h	Carbon emission intensity, carbon emission per unit GDP

**Table 14.** Change rate of each influencing factor (%).

Development scenario	time	P	g	e	c
Benchmark scenario	2010-2020	0.75	6.50	-2.90	-5.80
	2021-2035	0.6	6.00	-2.60	-5.20
	2036-2060	0.55	5.50	-2.40	-7.20
Natural scene	2010-2020	0.85	7.00	-2.20	-4.40
	2021-2035	0.80	6.50	-2.10	-4.20
	2036-2060	0.75	5.00	-2.00	-6.00
Ambitious scenario	2010-2020	0.75	6.00	-3.10	-6.20
	2021-2035	0.60	5.50	-3.00	-6.00
	2036-2060	0.55	4.50	-2.80	-8.40

**Table 15.** Target values of GDP, population and energy consumption.

time	GDP	Population (ten thousand)	Energy consumption
2025	114264.036	8850.725	36120.02
2030	138630.536	9140.07	39656.22
2035	162997.036	9429.415	43192.42
2050	236096.536	10297.45	53801.02
2060	284829.536	10876.14	60873.42

Analysis: weighting the results of the above correlation coefficient, and finally obtaining the correlation value, and using the correlation value to evaluate and sort the nine evaluation objects; The value of correlation degree is between 0 and 1. The greater the value, the stronger the correlation between it and the parent sequence, which means the higher its evaluation. As can be seen from the above table, for the nine evaluation items, the per capita carbon emission is the highest (correlation degree: 0.997), followed by the resident population (correlation degree: 0.994).

## Analysis of carbon emissions and regional human economy

(1) Energy consumption forecasting model based on population and economic changes.
  ① Use ARIMA model to predict the changes of population, GDP and energy consumption during 2021-2060.
  ② Establish a correlation model between population and energy consumption.
  ③ Establish the correlation model between economy (GDP) and energy consumption.
(2) Regional carbon emission prediction model
  ① Establish the correlation between carbon emissions and population, GDP and energy consumption.
  ② Establish the correlation between carbon emissions and energy consumption departments and energy supply departments.
  ③ Establish the correlation between carbon emissions and energy consumption varieties.

This paper includes establishing correlation and forecasting with ARIMA model. These models can help predict the changing trend of population, economy, energy consumption and carbon emissions in a certain region in the future, and provide reference for formulating low-carbon development strategies.

### (A) Based on population and economic changes in energy consumption forecasting model

Because GDP and population-related time t show a linear trend, it is preliminarily considered to establish a multiple linear regression model for prediction and analysis. The model formula is

$$y=ax_{1}+bx_{2}+c+\varepsilon$$

$$\varepsilon ∼\;\left(\mu ,\sigma^{2}\right)$$

In the previous analysis, both GDP and population have passed the normality test, so linear regression can be carried out. We use the least square method to determine the coefficient.

The result analysis of F-test shows that the P value of significance is 0.000***, which is significant horizontally, and the original hypothesis that the regression coefficient is 0 is rejected, so the model basically meets the requirements. For variable collinearity, variable GDP and VIF value of resident population are more than 10, and there is collinear relationship. The formula of the model is as follows: y (total energy consumption) =-0.001+1.0*GDP+0.001* resident population. Because the collinearity between variables is obvious, and r is 1, which shows that it is a functional relationship rather than a correlation relationship, the linear regression method should be abandoned, and the ridge regression method should be used to predict, and K=0.03 is determined according to the variance expansion factor method.

Analysis: The results of ridge regression show that the P value based on F test is 0.000***, which is significant at the horizontal level, rejecting the original hypothesis, indicating that there is a regression relationship between independent variables and dependent variables. At the same time, the goodness of fit r of the model is 0.996, and the model is excellent. The formula of the model: total energy consumption =-121268.079+16.388× resident population+0.782× GDP.

The prediction results of the model are as follows:

### (B) Regional carbon emission prediction model

A multivariate linear regression model can be established to predict carbon emissions and population, GDP and energy consumption. The result analysis of F-test shows that the P value of significance is 0.002***, which is significant horizontally, and the original hypothesis that the regression coefficient is 0 is rejected, so the model basically meets the requirements. The formula of the model is as follows: y=-71496.169+0.034*GDP+16.282* resident population+0.034* total energy consumption.

## 4. Carbon emission scenario analysis

### 4.1 Scenario design

According to the requirements of carbon emission policy, the following three scenarios are designed:

(1) Natural scenarios (without considering human intervention): the time nodes of peak carbon dioxide emissions and carbon neutrality are predicted.
(2) Benchmark scenario (on-time peak carbon dioxide emissions and carbon neutrality): Set the time nodes of peak carbon dioxide emissions and carbon neutrality, which are related to the improvement of energy efficiency and the increase of the proportion of non-fossil energy consumption.
(3) Ambitious scenario (taking the lead in peak carbon dioxide emissions and carbon neutrality): Set a more ambitious time node of peak carbon dioxide emissions and carbon neutrality, which is associated with the improvement of energy efficiency and the increase of the proportion of non-fossil energy consumption.

### 4.2 Multi-scenario carbon emission accounting method

Based on the basic assumptions, carbon emissions accounting:

Hypothesis 1: The GDP in 2035 will be doubled compared with the base period, and it will be quadrupled compared with the base period in 2060.

Hypothesis 2: The carbon consumption of ecological carbon sinks in 2060 is 10% of the carbon emissions in the base period. That is to say, the carbon emission intensity is reduced by 10%.

Hypothesis 3: In 2060, the carbon consumption of engineering carbon sinks or carbon transactions is 10% of the carbon emissions in the base period.

(1) Regional carbon emissions are consistent with the multi-scenario hypothesis.
(2) The regional carbon emissions are consistent with the total carbon emissions of various departments.
(3) The carbon emission accounting model is consistent with the prediction model in Question 2.

### 4.3 Determine the Double Carbon Target and Path

(1) Determine the target values of GDP, population and energy consumption (2025, 2030, 2035, 2050 and 2060).
(2) Determine the target values (2025, 2030, 2035, 2050 and 2060) for improving energy efficiency and increasing the proportion of non-fossil energy consumption.
(3) Complete qualitative and quantitative analysis of energy efficiency improvement, industrial upgrading, energy decarbonization and electrification of energy consumption.

Note: The change rate of influencing factors in the above table refers to the research results of Rui Qi (2023).

According to the report of World Economic Outlook 2019, the International Monetary Fund (IMF) confirmed by Kaya decomposition method that the decrease of global energy intensity and carbon emission intensity slowed down the growth of total carbon emissions from 2013 to 2017. However, the decrease in energy intensity in 2018 was small, which could not offset the increase in carbon emissions caused by population growth and per capita income growth, resulting in a 1% increase in global carbon emissions in 2017 and a 2% increase in 2018.

Since the 21st century, China’s influence on the growth of global carbon emissions has changed significantly. In 2015, coal consumption in China decreased by 3.7%. In the same year, China’s energy consumption reached 4.3 billion tons of standard coal, and the goal in 2020 is to limit it to 5 billion tons. The government of China has set mandatory targets, including air pollution control and strengthening the development of renewable energy such as wind, solar and nuclear energy.

According to the preliminary accounting of the National Bureau of Statistics, the total energy consumption in China in 2020 is 4.98 billion tons of standard coal, accounting for 56.8% of the total energy consumption, down 0.9 percentage points from the previous year. According to Tollefson, China may reach the peak of total carbon emissions earlier than expected. However, the power demand in China will continue to grow at a high speed, so it is necessary to promote the decarbonization process of the power industry to speed up the reduction of carbon emission intensity.

**Three situations in peak carbon dioxide emissions:**

**Figure 1.**
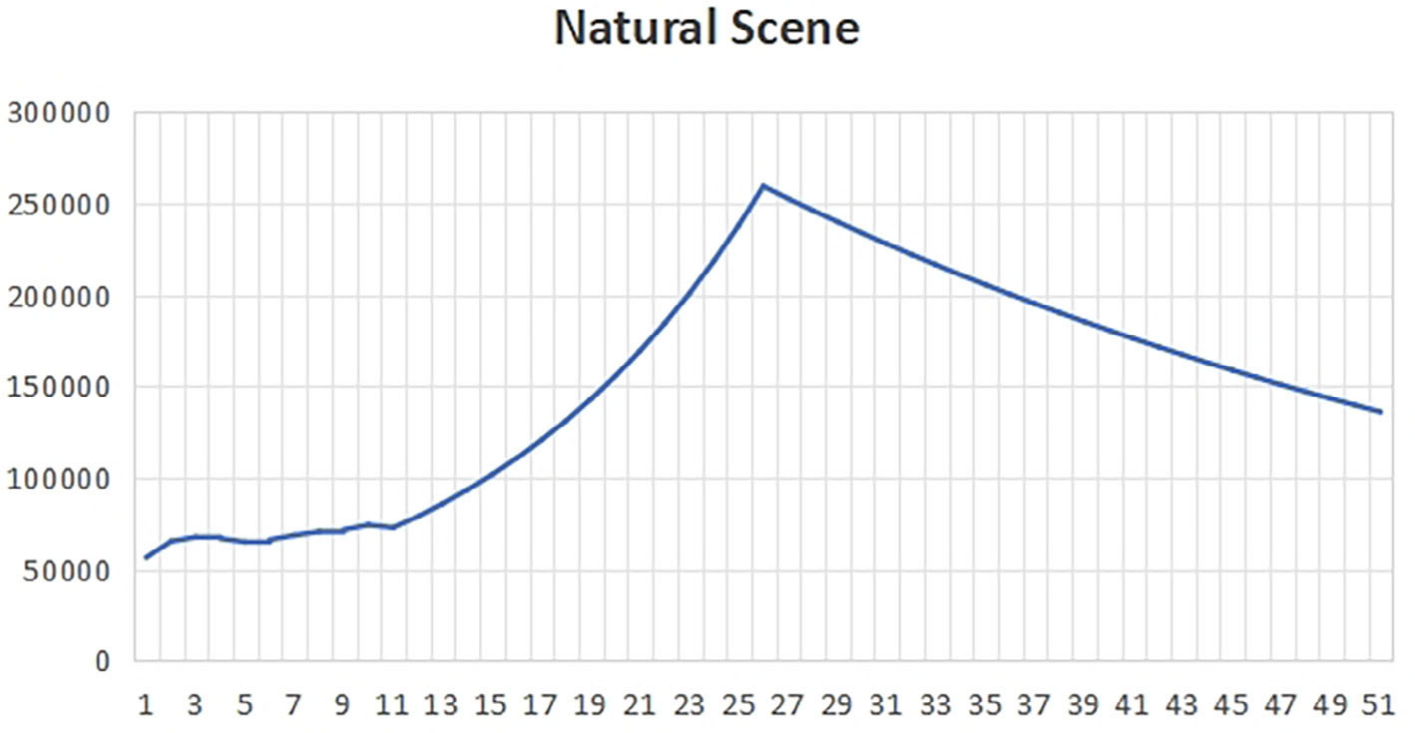
Total carbon emissions in baseline scenario.

**Figure 2.**
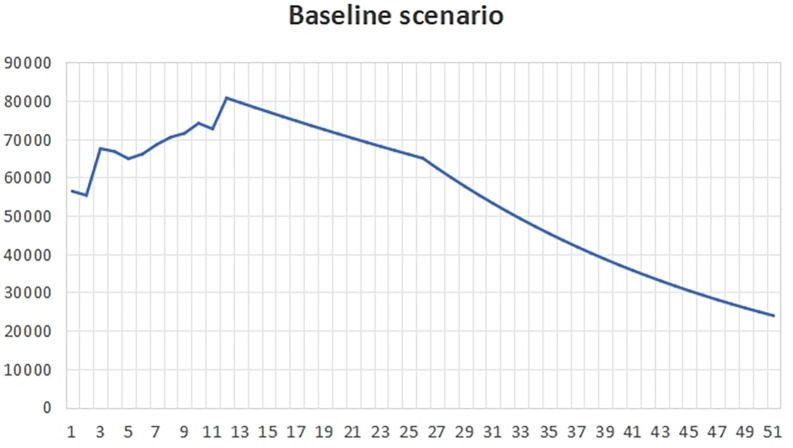
Total carbon emissions in natural scenarios.

**Figure 3.**
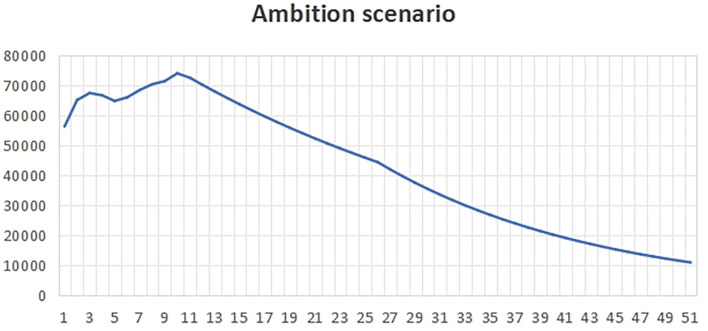
Total carbon emissions in ambitious scenarios.

(1) The benchmark scenario
  It can be seen from the Figure 1 that the total carbon emissions in the baseline scenario will reach the peak around 2022, that is, the life span of peak carbon dioxide emissions is 2022, which is about 8 years earlier than the expected year of 2030. This is because all indicators in the baseline development scenario meet the minimum standards required by the government. At this time, the policy will pay attention to the impact on the ecological environment, and it is more likely to develop the economy while taking into account the environment.
  In the years after peak carbon dioxide emissions, we can see that the total carbon emissions began to decline gradually, which shows that policy makers and all walks of life have begun to actively respond to climate change and take effective measures to reduce carbon emissions. With the continuous progress of technology and the vigorous promotion of clean energy, more enterprises and individuals began to use low-carbon, environmentally friendly products and services, thus reducing the damage and pollution to the environment.
  In the baseline scenario, the government will strengthen supervision and law enforcement, and promote the management and emission reduction of carbon emissions in various industries. At the same time, the government will also increase the research and development and promotion of clean energy and low-carbon technologies, encourage enterprises to adopt more environmentally friendly production methods, and promote the development of green economy.
  In addition, the government will also strengthen public education and publicity on environmental protection, and raise public awareness and awareness of climate change and environmental protection. Through the joint efforts of the whole society, we can expect to achieve a significant reduction in carbon emissions in the next few decades, so as to achieve the goal of carbon neutrality and protect our home on earth.
(2) Natural scenes
  Under the natural development scenario Figure 2, the change rate is higher than the benchmark value, reflecting that the local government pays more attention to economic development and less attention to carbon emissions. At the same time, it reflects that the government has less macro-control and more natural ways to prevent carbon emissions from economic development.
  The natural time in peak carbon dioxide emissions is 2036, which is about 6 years later than the expected peak carbon dioxide emissions time.
(3) Ambition scenario
  Under the ambitious development scenario Figure 3, our goal is to take long-term development as the guide, and at the same time, on the basis of taking into account the ecological environment and economic development, realize harmonious symbiosis. In order to achieve this goal, we strictly control the change rate of each influencing factor to ensure that it is lower than the benchmark value.
  The peak carbon dioxide emissions time of the ambitious scenario is around 2021, which is 9 years earlier than the expected peak carbon dioxide emissions life.
  In order to advance carbon emissions, the following ambitious initiatives are needed:
  First of all, in terms of ecological environment, we pay attention to protecting natural resources, reducing pollution emissions and improving energy efficiency. This means that we should strengthen the control of environmental pollution, improve environmental quality and create a livable living environment for the people. At the same time, we should promote green development, encourage the development of green industries and advocate a low-carbon lifestyle in order to realize the sustainable development of the ecological environment.
  Secondly, in terms of economic development, we should adhere to the development concept of innovation, coordination, green, openness and sharing. This means that we should improve the quality of economic growth, promote the optimization and upgrading of industrial structure, and give play to the basic role of advanced manufacturing and modern service industries. In addition, we must expand the international market, strengthen economic cooperation with other countries in the world, achieve mutual benefit and win-win, and improve China’s position in global economic governance.
  In this process, we should give full play to the positive role of the government, enterprises, social organizations and the public. The government should fulfill its responsibilities of macro-control, market supervision, public service and social management, and create a good development environment for enterprises. Enterprises should assume social responsibilities, strengthen self-discipline, improve management level and provide good welfare for employees. Social organizations should actively participate in social affairs and provide public services to meet the diverse needs of the people. The public should establish correct values, actively participate in social construction, promote positive energy and contribute to the long-term development of the country and the nation.
  In short, under the ambitious development scenario, we should take long-term development as the goal, take into account the ecological environment and economic development, and strive to achieve harmonious coexistence between man and nature and between people. This requires us to work together, innovate constantly, make positive progress, and work hard to build a better future.

## 5. Conclusion and prospect

According to the research results, we put forward the following suggestions:

First of all, we need to re-understand the relationship between economic growth and carbon emissions, and firmly promote the process of China’s carbon emissions peaking. Although some people think that limiting carbon emissions will harm economic growth, in the long run, carbon emissions will gradually reach the peak with economic growth. Therefore, we should not unilaterally think that carbon emission constraints will definitely harm economic growth, but should form a unified consensus on emission reduction and promote high-quality social and economic development.

Secondly, we should focus on cultivating new kinetic energy for sustainable economic growth. The EU has formed a leading advantage in technological and institutional innovation in developing energy-saving technologies, improving energy efficiency, changing energy structure and increasing the proportion of renewable energy. By promoting the energy revolution and industrial technological innovation, it is possible to create a scientific and technological revolution and promote the new glory of economic transformation. We need to change our consciousness and take a better look at the development of new technologies and their benefits. Intelligent and networked large-scale computing is the foundation of innovation and will continue to promote innovation.

Finally, we should improve the quantifiable policy system for different fields. The carbon emission policy design of governments in different fields, such as carbon tax or carbon emission quota trading, has a quantifiable policy effect on long-term growth. For example, the European Union has skillfully implemented the strategy of linking carbon tax with carbon emission price, and implemented the policy of building energy conservation and emission reduction, which is closely linked with the European Union’s carbon emission trading system and has achieved remarkable results. In promoting the healthy development of the carbon emissions trading market, the United States has adopted a series of well-designed tax policies, which not only enhanced the activity of the carbon trading market, but also ensured the fairness of the carbon emitters in taxation. Therefore, we can learn wisdom from these successful experiences and turn them into strategies suitable for our national conditions, so as to build an institutional mechanism that is more conducive to carbon emission peaking and carbon neutrality.

## 6. Research limitations

Although this study conducted an in-depth analysis of carbon emissions in specific regions under the “dual carbon” target by constructing a grey prediction autoregressive model based on entropy weight method, and proposed corresponding policy recommendations, there are still some limitations that may affect the comprehensiveness and accuracy of the research results.

### 6.1 Data limitations

The data for this study mainly comes from public channels such as the International Energy Agency (IEA). Although these data are authoritative, their timeliness and completeness may be limited to some extent. Especially for carbon emission data specific to certain regions or sub sectors, there may be missing or inaccurate data, which to some extent affects the accuracy of the analysis. In addition, the collection and organization of carbon emission data involves multiple departments and fields, and the consistency and comparability of the data may also be affected, which may lead to potential biases in the research results.

### 6.2 Limitations of the method

This study used various methods such as entropy weight method, grey prediction, and autoregressive model. Although these methods have certain advantages in dealing with complex systems, they also have limitations. For example, grey prediction models are more suitable for situations with limited data and incomplete information, while their prediction accuracy may not be as good as other more complex models when there is sufficient data. In addition, although the entropy weight method can objectively determine the weights of indicators, it has certain requirements for the quality and distribution of data. Abnormal or missing data may lead to unreasonable weight allocation, which in turn affects the stability and reliability of the model.

### 6.3 Assumptions and model limitations

When constructing the model, this study made some assumptions, such as simplifying the impact of economic growth, population growth, and changes in energy consumption structure on carbon emissions as a linear relationship, and ignoring the interactions between various factors. However, the actual situation may be more complex, and nonlinear relationships and interactions between factors may have significant impacts on carbon emissions. In addition, the selection and setting of model parameters may also be influenced by subjective factors, which to some extent increases the uncertainty of prediction results.

### 6.4 External validity

This study mainly analyzes specific regions, and its research results may not be applicable to other regions or larger areas. There are differences in economic development level, energy consumption structure, policy environment and other factors in different regions, which may significantly affect the trend and influencing factors of carbon emissions. Therefore, when extending the results of this study to other regions, it is necessary to carefully consider these differences and make corresponding adjustments and validations.

### 6.5 Limitations of practical application

Although this study proposes a series of policy recommendations, these recommendations may face many challenges in practical application. For example, policy implementation requires cooperation and support from multiple parties such as the government, enterprises, and the public, and the demands and interests of different stakeholders may conflict, which may increase the difficulty of policy implementation. In addition, the effectiveness and sustainability of policies also need to be verified through long-term practice, which to some extent limits the immediate impact and application scope of policies.

## Ethics and consent

Ethical approval and consent were not required.

## Author contributions

Sanglin Zhao: Conceptualization, Data curation, formal analysis, Software, Writing–original draft, Visualization, Writing–review and editing;Hao Deng: Conceptualization, Data curation, formal analysis, Software, Writing–original draft, Visualization, Writing–review and editing Jackon Steve: Validation, Writing review and editing. All authors have read and approved the final version of the manuscript.

## Software availability description

The software used in this study is all free and publicly available genuine software. WPS Excel (or office Excel can also replace it) is used for data processing, and WPS Excel is used for image drawing.

## Data Availability

Figshare: Research on Statistical Measure under Double Carbon Target-Self-moving regression model of grey prediction based on entropy weight method, https://doi.org/10.6084/m9.figshare.28524602.v1. ^26^ This project contains the following underlying data:
1. Data 1. xlsx 2. data 2. xlsx 3. result data.xlsx Data are available under the terms of the Creative Commons Zero (CC0) “No rights reserved” data waiver Figshare: Research on Olympic medal prediction based on GA-BP and logistic regression model checklist, https://doi.org/10.6084/m9.figshare.28374260.v4. ^27^ This project contains the following underlying data:
1. checklist.docx 2. STROBE-checklist-v4-combined.doc 3. Processed data.xlsx 4. data_dictionary.csv 5. summerOly_programs.csv Data are available under the terms of the Creative Commons Zero (CC0) “No rights reserved” data waiver CO2 Emissions in 2023 – Analysis - IEA, https://www.iea.org/reports/co2-emissions-in-2023, IEA. Part of the research data in this study comes from publicly shared carbon emission data by the International Energy Agency. Readers or reviewers can access all necessary information required for the data in the same way as the author. ( https://www.iea.org/reports/co2-emissions-in-2023)
