# Supplementary material for: Research on statistical measure under double carbon target - Self-moving regression model of grey prediction based on entropy weight method
Source: F1000Res. 2025 May 6;14:437. Originally published 2025 Apr 15. [Version 2] doi: 10.12688/f1000research.162696.2 (PMC12417981; doi:10.12688/f1000research.162696.2)
Supplement: Peer review history

# Reviewer report: Reviewer response for version 2

**DOI:** 10.5256/f1000research.181498.r407024

**Published:** 2025-09-08

**Authors:** Ming-Feng Ge

**Affiliations:** 1 China University of Geosciences Faculty of Engineering, Wuhan, Hubei, China

This manuscript has studied a comprehensive indicator system and relationships among these factors. The prediction of the future carbon emissions was provided and propose suggestions for dual carbon path planning. The following comments should be taken into account.

 1. The abstract should indicate specific methods for analyzing the main factors of the study。

 2. The content and structure in the INTRODUCTION section should be optimized, such as discussing the research background and significance first, and then introducing the research method of this manuscript.

 3. In the INTRODUCTION section, the cited literature should be related to the method proposed in this manuscript, while the existing work should be analyzed and summarized, and the main contribution should be pointed out to highlight the solved problems and innovations of this manuscript.

 4. In section II, the relationship between consumption in various social departments and the selected indicators should be supplemented.

 5. In section II, more detailed analysis about correlation among indicators should be supplemented to support subsequent analysis.

 6. It is mentioned in the abstract that Kaya method and various factor models were used to predict carbon emissions, and the description of these methods should be added in this manuscript.

 7. The specific content of the manuscript should be carefully checked, there is a lack of necessary content in some places, such as the lack of specific content after the subsection of Trends of indicators in subsection 2.4, and others problems including the wrong position of the variable symbol, the inconsistency of graphics and text, and some of the writing is not clear and coherent.

Is the work clearly and accurately presented and does it cite the current literature?

Yes

If applicable, is the statistical analysis and its interpretation appropriate?

Partly

Are all the source data underlying the results available to ensure full reproducibility?

Yes

Is the study design appropriate and is the work technically sound?

Yes

Are the conclusions drawn adequately supported by the results?

Yes

Are sufficient details of methods and analysis provided to allow replication by others?

Yes

Reviewer Expertise:

Research Interest:Systems and control theory as well as their applications to autonomous and robotic systems, networked Systems, cyber-physical systems, artificial intelligent systems, power grids.

I confirm that I have read this submission and believe that I have an appropriate level of expertise to confirm that it is of an acceptable scientific standard, however I have significant reservations, as outlined above.

# Reviewer report: Reviewer response for version 2

**DOI:** 10.5256/f1000research.181498.r407023

**Published:** 2025-08-25

**Authors:** Xuefeng Jiang

**Affiliations:** 1 East China Normal University Department of Education, Shanghai, Shanghai, China

The study focuses on the statistical measurement of carbon emissions in specific regions under the dual-carbon goals. It employs a grey prediction autoregressive model based on the entropy weight method to analyze the relationships between carbon emissions, the economy, population, and energy consumption, and forecasts the timing of carbon peaking under different scenarios. The research problem carries clear practical significance and policy value, the methodological combination is relatively innovative, the data sources are authoritative, and the analytical process is detailed. The conclusions provide useful references for policy-making. The article has a complete structure, clear logic, and generally standardized language expression. However, several questions still need to be answered and issues need to be improved. This reviewer recommends its indexing after the following revision.

 1) The coverage of the latest research progress (especially from 2023–2024) is insufficient. It is recommended to supplement recent studies on carbon peaking pathways, regionally differentiated policies, and international comparisons to enhance the timeliness and depth of the literature review.

 2) The study assumes a linear relationship between factors such as economic growth and population with carbon emissions, overlooking possible nonlinear interaction effects.

 3) The selection of models and indicators is reasonable and applicable at the framework level, but the methodological execution could be further elaborated and more deeply justified.

 4) Data timeliness and regionality: the IEA data used is from 2023, while the study projects up to 2060. How can the uncertainty of such long-term forecasts be ensured? Should sensitivity analysis be conducted for key parameters?

 5) Policy recommendations are somewhat generalized. The proposed suggestions are rather conventional and lack specific implementation pathways.

 6) Formatting issues: numbers and units, as well as spacing before and after punctuation marks, are often inconsistent. The reference list format should also be standardized, for example, whether article titles should be uniformly bolded, whether author names should follow a unified style, and whether journal page numbers need to be checked and presented in a consistent format.

Is the work clearly and accurately presented and does it cite the current literature?

Yes

If applicable, is the statistical analysis and its interpretation appropriate?

Yes

Are all the source data underlying the results available to ensure full reproducibility?

Yes

Is the study design appropriate and is the work technically sound?

Yes

Are the conclusions drawn adequately supported by the results?

Partly

Are sufficient details of methods and analysis provided to allow replication by others?

Yes

Reviewer Expertise:

Recycling

I confirm that I have read this submission and believe that I have an appropriate level of expertise to confirm that it is of an acceptable scientific standard, however I have significant reservations, as outlined above.

# Reviewer report: Reviewer response for version 2

**DOI:** 10.5256/f1000research.181498.r383097

**Published:** 2025-08-09

**Authors:** Devarakonda. Sameer Kumar

**Affiliations:** 1 QIS College of Engineering and Technology, Guntur, India

I have no further comments.

Is the work clearly and accurately presented and does it cite the current literature?

Yes

If applicable, is the statistical analysis and its interpretation appropriate?

Yes

Are all the source data underlying the results available to ensure full reproducibility?

Yes

Is the study design appropriate and is the work technically sound?

Yes

Are the conclusions drawn adequately supported by the results?

Yes

Are sufficient details of methods and analysis provided to allow replication by others?

Yes

Reviewer Expertise:

I have published papers on the areas of MADM Techniques , Entropy weighting method , Mean Weighting Methods,  Geometric mean Methods, Taguchi and GRA Analysis

I confirm that I have read this submission and believe that I have an appropriate level of expertise to confirm that it is of an acceptable scientific standard.

# Reviewer report: Reviewer response for version 1

**DOI:** 10.5256/f1000research.178937.r381269

**Published:** 2025-04-30

**Authors:** Devarakonda. Sameer Kumar

**Affiliations:** 1 QIS College of Engineering and Technology, Guntur, India

The work is good but needs minor modifications.

 1. The authors can extend more literature to support the novelty of problem and methodologies provided.

 2. To help other readers, the authors should explain how their proposed model achieved the maximum yield.

 3. Since different models propose , different weights , how the solutions are robust..? have you conducted any sensitive analysis.

 4. Specify the reasons , why you have modelled the problem as liners.

 5. Please indicate the tabular values in same format through out the paper. Some has two decimals and and some has three decimals.

 6. The data provided in the tables is discussed less.

Is the work clearly and accurately presented and does it cite the current literature?

Yes

If applicable, is the statistical analysis and its interpretation appropriate?

Yes

Are all the source data underlying the results available to ensure full reproducibility?

Yes

Is the study design appropriate and is the work technically sound?

Yes

Are the conclusions drawn adequately supported by the results?

Yes

Are sufficient details of methods and analysis provided to allow replication by others?

Yes

Reviewer Expertise:

I have published papers on the areas of MADM Techniques , Entropy weighting method , Mean Weighting Methods,  Geometric mean Methods, Taguchi and GRA Analysis

I confirm that I have read this submission and believe that I have an appropriate level of expertise to confirm that it is of an acceptable scientific standard, however I have significant reservations, as outlined above.
